
JOURNAL INFORMATION
==============================
NLM Title Abbreviation: Med Chir Rev
NLM Journal ID: 0034617

The Medico-Chirurgical Review

Publisher: Biomedical Journal Digitization Project

ARTICLE INFORMATION
==============================
PMCID: PMC4966247
PMID: 29917619
Article version: 1
Subjects: Reviews, Diseases Produced by Malaria

New Method of Treating Artificial Anus

Print publication date: 1828 Oct 1
Volume: 9
Issue: 18
First page: 313
Last page: 323
1828 Jul
License: This work is free of known copyright restrictions. For more information, please see PMC Back Issue Digitization.
License URL: https://creativecommons.org/publicdomain/mark/1.0/

==============================



### IV.

### *New Method of treating Artificial Anus.* By Baron Dupuytren.*

M. Dupuytren has published a long and interesting paper on the cure of that most disgusting and dangerous malady, artificial anus. The affection, or rather affliction, has been hitherto considered as incurable, but we are assured by the able author of the Memoir, that, after the details which he has given, and the treatment which he recommends, it will be ranked with those diseases which, with little difficulty or danger, admit of relief from art. This is so encouraging a prospect, a consummation so devoutly to be wished for, that we betake ourselves, *con amore*, to the drudgery of analysis and abridgement.

The Baron commences his Memoir, with a series of preliminary observations, the object of which is to demonstrate, that different portions of the alimentary canal play different parts in the elaboration of the chyle, and that, if the natural condition of the tube be altered or disturbed, that very important office will be altered and disturbed also. These are very obvious truths, and we shall pass them over.

The preternatural opening of the intestines is rarely congenital, but mostly results from wounds, inflammations, abscesses, or hernia, and especially the latter, to which the Baron confines his observations.

The establishment of an artificial anus is by no means so simple a process as would, at first sight, be supposed. The intestine which is to form the anus, must be opposite that part of the parietes of the abdomen through which the matters are to make their exit, and it must be retained in this situation, or eventually fixed in the opening; a ready communication must be maintained between the external aperture and the bowels; and, above all, these last must be capable of adhering to the surrounding parts, a combination of circumstances which experience has shewn to be rare. The new anus, when established, is generally round, but occasionally irregular; from a few lines to an inch or more in diameter; and surrounded by radiating plaits of the integument. The cicatrix of the border unites the skin of the belly to the mucous membrane of the bowel, whilst the intestine and abdominal parietes are joined together by inflammatory adhesions, which always commence in the serous surface of the intestine and abdominal cavity, and extend to the other textures, the skin and the mucous membrane. Their extent is, in general, from half a line to a line, sometimes several, and rarely half an inch. The connecting medium is first glutinous, then cellular, and, at last, of a fibrous structure, when it is firm enough effectually to resist those causes which tend to separate the bowel from the walls of

* Mémoires de l'Académie Royale de Médécine, Tome I.

the abdomen. As the adhesions extend only a small distance along the gut, a *cul-de-sac* is formed, the opening looking towards the belly, into which the abdominal viscera may protrude, and complicate, more or less, the artificial anus.

The opening of the anus is mostly occupied by the puckered internal membrane of the bowel, which not unfrequently protrudes, and proves a source of inflammation to the mucous membrane. The eversion varies in length from one to fifteen inches, or even more, and generally occurs from the upper end of the intestine, sometimes from the lower, occasionally from both.

Between the opening of the skin and the bottom of the artificial anus is a funnel-shaped cavity, of which the skin forms the border, and the intestine the base, and which varies in length, direction, and dimensions. In proportion as these dimensions are greater, the tendency in the disease to be cured by nature or by art is greater also. At the bottom of this cavity are the orifices of the two extremities of the intestine, the upper of which is much the larger, and the partition which divides them. The extremities of the intestine, to which the orifices lead, are villous, covered with mucous secretions within, and serous without, and retire into the abdomen, sometimes crossing and sometimes parallel, but generally separating at an angle more or less acute. The partition between the orifices is produced by the juxta-position and adhesion of the sides of the intestines, and juts out nearer to, or farther from the skin, in proportion as the intestine has suffered a greater or less loss of substance from mortification, or undergone a more or less considerable change in its situation. When the gut has been merely pierced by a wound or eschar, it is small, and scarcely to be seen, so that there exists between the two orifices of the bowel a kind of gutter, directing the transit of matters from the upper to the lower, and greatly facilitating the attempts at cure. When, however, the whole circumference of the intestine has been destroyed, it is very great, and forms a projecting angle or buttress between the ends of the intestine, which its contents can neither break down nor get round.

This *buttress*, examined from the cavity of the intestine, has a semilunar shape, but, viewed from within the belly, it is seen to unfold itself, and the two equal parts of which it is composed, receive the mesentery between them. This results from its consisting at every point, except its acute edge, of two sides, having a triangular interval between them, which grows larger, in proportion as they separate from each other on entering the abdomen. The surfaces of this double partition towards the belly are smooth and unadherent, so that, to pass from one extremity of the intestine, through the partition, to the other, it is necessary to traverse the peritoneal cavity, a circumstance attended, of course, with considerable danger.

The buttress and double partition are attached to the mesentery, and follow, to a certain extent, the movements communicated to them by that ligament.

The mesentery itself, stretching from the vertebræ to the concave side of the intestinal convolutions, is always more or less dragged on when the intestine is in any way protruded from the belly, in which case it forms a kind of cord from the vertebral column to the part of the bowel most distant from it. This cord is necessarily tense, and inclines the body forward, as is particularly observed in adherent herniæ. In the artificial anus, the intestine is constantly pulled inwards by the mesentery, in proportion to the tension of the membrane; and in two cases M. Dupuytren has known it sufficient to destroy the adhesions between the bowel and walls of the abdomen, causing fatal laceration, and effusion into the cavity of the peritoneum. From this, it is obvious that position, and the movements of the body, must have a considerable influence on the cure of the disease.

The action of the mesentery is continued long after the malady is removed. Several patients who had been cured of artificial anus were re-admitted into the Hôtel Dieu, and died of other diseases, when dissection shewed the intestine, not adherent to the walls of the abdomen, but free and unattached, a fibrous cord, however, still continuing stretched between them. This last was so attenuated at its middle, that its laceration would probably have soon destroyed even the last trace of the derangement which had attended the formation of the artificial anus.

Of the two extremities of the intestine divided by the intermediate partition, the upper is by far the larger, because its activity is increased by the presence and passage of the intestinal contents, a change in which the mesentery and mesenteric glands participate. The lower portion not performing its natural functions, becomes attenuated, though not entirely obliterated, as it is filled to a certain extent with a soft, white mass, of albuminous appearance, which is an imperfect intestinal secretion, and may remain undecomposed for months, or years, till it is either voided by stool, or washed out by injections. The orifice of this lower portion of the bowel is generally narrow, puckered up, and difficult to find, from the buttress being thrown down upon it by the passage of the fæculent matters from above, and acting as a kind of valve.

Another consequence of the disease is the loss of the natural mobility of the gut, which is absolutely fixed by the new adhesions. The fixed portion thus becomes a *point d'appui* for the efforts of the canal, so that the fæces are constantly directed towards it, and their progress from the stomach downwards actually accelerated. The space traversed by the aliments is shortened, as well as the period of their detention—their digestion is incomplete—nutrition is impaired, according as the anus is farther or nearer from the stomach, and the evacuation of the bowels is no longer voluntary. In the original is a lithographic plate, representing the parts of which the foregoing anatomical description has been given. To make that description intelligible, we shall introduce a wood-cut here, copied from the original lithograph, but on a smaller scale.

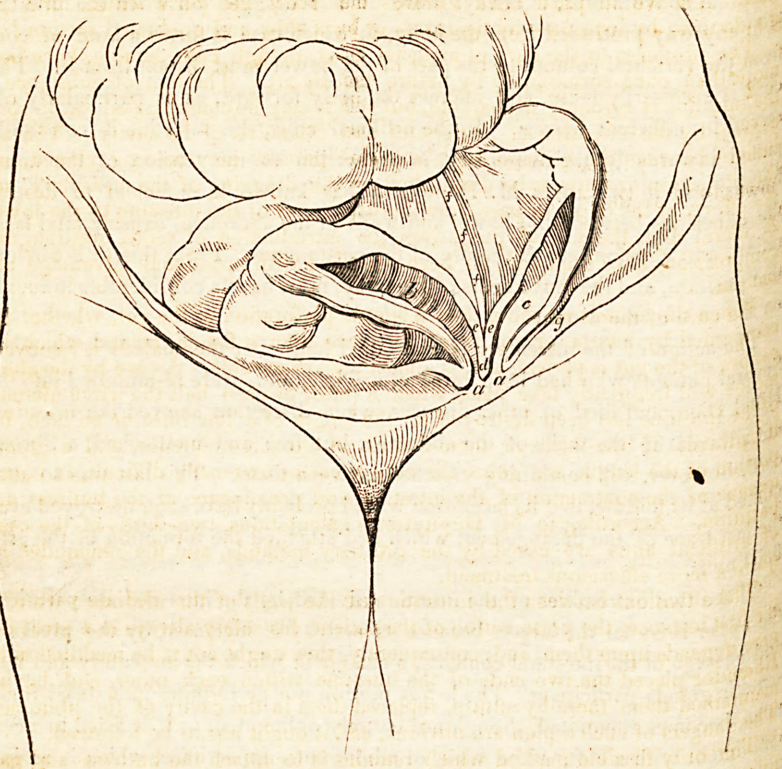

### EXPLANATION OF THE WOOD-CUT.

*(a)* Opening of the artificial anus, and point of union between the mucous membrane and the skin.

*(b)* Upper end of the intestine.

*(c)* Lower end of the intestine.

*(d)* Buttress, formed by the union of the walls of the two intestines.

*(ee)* Walls of the intestine, forming the "double partition."

*(f)* Cord or ligament formed by the mesentery.

*(g)* Infundibulum or *cul de sac*, between the peritoneum of the intestine and of the abdominal parietes, into which herniæ occasionally protrude.

With regard to this involuntary evacuation, the opening is not surrounded by any sphincter, and is, therefore, always open to the matters which are constantly coming up. If there were a sphincter, still there would be no reservoir, like the large intestine, and, consequently, there would still be a constant flow of mucous, biliary, alimentary, or fæculent matters, according to the state of the digestion and situation of the opening, affecting the person of the patient with an abominable smell, and disposing the parts to excoriations, erysipelas, and an intolerable itching, which renders life a burthen. Compression, so as to retain the matters within the bowel, has been tried, but often given rise to the greatest mischief. The projection, more or less, of the buttress which divides the extremities of the intestine, is evidently the cause which opposes or facilitates the cure, and the mode in which this obstacle can be overcome is a serious question. Are we to push back towards the belly the parts which form it? divide them by incision—by ligature—or by a slow and graduated section?

The obstacles to the cure of artificial anus, though considerable, have been more than once surmounted by nature and by art. The loss of substance in the intestine is certainly irreparable, but still it may be compensated for, in some degree, by the extension of its parietes and dilatation of its cavity. The adhesions between the gut and the abdominal parietes may become relaxed, and the projection or buttress be diminished by the dragging of the mesentery, and the efforts of the fæces to pass from the upper end of the intestine to the lower. A liberal diet, the use of purgatives, the introduction of tents, position, and compression, have each and all been attended with occasional advantage and success.

When the anus is no more than a simple perforation of the gut, whether accompanied by hernia or not, it is little more than a fistula, behind which the calibre of the gut is perfect, and is almost always curable. A cure by the above means will frequently take place when a third, or even half the circumference of the intestine has been destroyed for the length of a few lines or an inch, but if upwards of two-thirds or three-fourths of the circumference, and a greater portion of the length, are gone, the cure becomes extremely difficult, in consequence of the contraction of the intestine, and prominence of the buttress and partition. According to M. Dupuytren's calculations, two-thirds of the cases of artificial anus are cured by the ordinary methods, and the remainder require a more efficacious treatment.

The adhesions between the intestine and the walls of the abdomen were, in the first instance, the preservation of the patient, his safety always in a great degree depends upon them, and, consequently, they ought not to be meddled with. Rhamdor placed the two ends of the intestine within each other, and, having maintained them there by suture, replaced them in the cavity of the abdomen. The dangers of such a plan are obvious, and it ought not to be followed.

The only feasible method which remains is to attack the buttress and partition, which prevent the flow of fæces through the tube, and direct them to the artificial anus. A simple section of the projection by the scissors or other cutting instrument, would most probably be attended with an almost immediate death, from a passage being opened for the fæcal matter into the cavity of the abdomen. On this account, division by a cutting instrument was out of the question; but it seemed more reasonable, and certainly more safe, to push back the projection by pressure from without, so as to imitate, in some measure, the dragging of the mesentery from within. M. Dupuytren constructed an instrument, which he tried upon a patient at that time (1809) in the Hôtel Dieu, but colic and nausea were produced, and the attempt was given up.

The idea of perforation next presented itself, but still the peritoneum, which surrounds the two extremities of the gut which forms the artificial anus, would be wounded, and effusion of the fæcal matters would result. One of the most remarkable properties of serous membranes, when inflamed, is to contract adhesions, and M. D. imagined that, if he could get the two surfaces of the gut to adhere together, he might afterwards perforate them with perfect safety.

The passage of foreign bodies, as pins and needles, through the parietes of the abdomen or intestines, is always preceded by the adhesive inflammation, and our author conceived that he might, in the same manner pierce the partition by a needle, armed with a thread. This would fill the void which had been made, and, having excited a degree of inflammation around it, might be gradually increased in size, until large enough entirely to destroy the partition. With the view of confirming his opinions by experiment, M. D. passed needles, armed with threads, through the intestines of several dogs, replacing the whole in the abdomen. Adhesive inflammation followed, the ligatures were either voided by stool, or taken away by gently pulling both ends at once, and in not a single instance did effusion occur. An artificial anus was even made in one dog, with the same results.

In 1813, a man who had laboured under strangulated hernia for five days, was admitted into the Hôtel Dieu with an artificial anus. Pressure was employed, but it gave rise to severe symptoms, and was abandoned. At the expiration of six weeks, the cure had not advanced, and the patient was very anxious that something should be done. Accordingly, M. Dupuytren passed a needle across the buttress and partition, which projected very considerably, and, having withdrawn the needle, allowed the thread to remain. No unpleasant symptom was the consequence, and, some days afterwards, a skein was substituted for the thread, when flatus began to be discharged by the natural anus. The size of the skein was increased each dressing, colicky pains occurred, and some fæces were passed by the anus. The size of the skein was increased so far, that one day the buttress was completely lacerated, but without any ill effect. Stercoraceous matter continuing to be voided by the artificial anus, M. Dupuytren divided, half a line at a time, by means of blunt-pointed scissors, directed on the fore-finger, those parts of the partition situated above the opening made by the thread. The incisions were cautiously made at intervals of three or four days, the adhesions that had formed were never passed, and the communication was soon so free, that the fæces passed entirely by the natural anus. Compression on the artificial opening was then employed, but the patient urging the surgeon to hasten the cure by some fresh attempt, several projections around the aperture were tied and excised, and then the partition divided higher than it had yet been done. In a few hours, acute peritonitis supervened, and the patient died. On dissection, the evidences of inflammation were discovered, but no effusion or opening into the cavity of the abdomen. The communication between the extremities of the gut was re-established for the space of two inches, and there was every reason to believe that, but for the peritonitis, the disease would have been cured.

On consideration, M. Dupuytren was determined to abandon the employment of the needle. In the above case it produced no effusion, yet, by penetrating the parts before adhesion is produced, it might be attended with that result in others. It would also be extremely difficult, by the above means, to divide the partition at such a height, as in every case to open a communication sufficiently free between the two ends of the intestine; and, finally, if the sides of the gut were separated when the needle pierced them, adhesion could not be produced, but, instead of it, a very dangerous communication with the cavity of the abdomen. On these accounts, the needle was abandoned.

The point appeared to be, to keep the parts in contact till adhesion had occurred, and then to effect their division, but not before. After many trials on animals, and the dead body, M. Dupuytren thought he had discovered the instrument he sought for. It consists of a screw and two branches, each about seven inches long. One branch, which may be called the male, being received into the other, has a blade four inches in length, three lines in breadth, and half a line in thickness at its edge, which is undulated, and terminated by a spheroid button. At the union of the blade with the handle is a mortise some lines in extent; the handle itself is one, two, or more inches, and has another mortise, three or four lines broad, which runs nearly the whole of its length. The female branch is not quite so long. It is formed at one of its extremities of two blades, of the same length, breadth, and thickness, as the small blade, and between the two is the kind of gutter or sheath in which the small blade is received, whilst the button of the latter is received into a cavity at the corresponding extremity of the former. At the junction of the blade with the handle is a moving pivot, which passes into the mortise of the other branch, and the handle itself is terminated by a hole to receive the screw.

This last part of the instrument, a screw of several threads, is an inch and a half in length, and terminated by an oval plate; it is placed in the mortise of the male branch, and fixed in the female, its use being to separate or close at pleasure the two blades of the *enterotome*, the name which is given to the instrument.

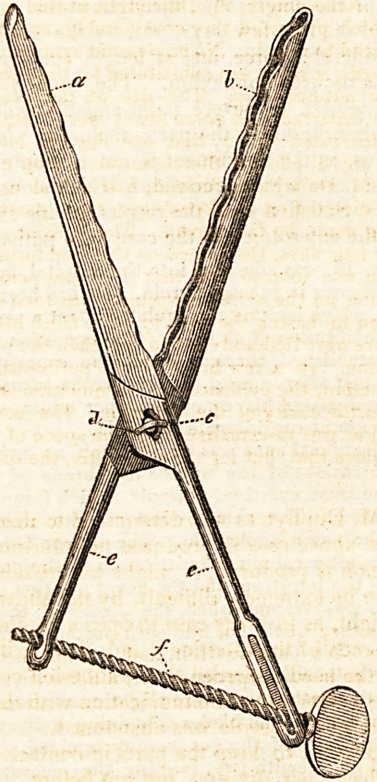

EXPLANATION OF THE WOOD-CUT.

*The Cut represents the Enterotome open.*

(*a*) The male blade.
(*b*) The female blade.
(*c*) The joint.
(*d*) The moving pivot.
(*e*) The handles.
(*f*) The screw by which the handles, and, in consequence of their crossing, the blades, likewise, may be approximated.

The application of it is as follows. The two branches, which may be separated or united at pleasure, provided with blades, and undulating and very blunt edges, are put in motion by means of the screw which passes across their handles. Whatever the blades inclose, is grasped and retained by them, partly by means of their denticulated edges, partly from the one being received within the other. At first, the parts inclosed are merely kept in contact by the pressure, but this may be afterwads so far increased as to destroy their vitality, though not immediately divide them, on account of the bluntness of the edges. Before employing the instrument on the human subject, M. Dupuytren made experiments on animals, in whom he always succeeded in dividing the parts inclosed in six or eight days. When serous membranes were confined, both they and the parts invested by them had united by the second or third day, whilst the solution of continuity never occurred till the seventh or eighth.

The adhesions, so necessary to the safety of the patient, were continued on each side of the branches of the enterotome, throughout their whole extent, were easily destroyed during the first few days, but, by the sixth or seventh, were so firm as to resist considerable force, and at length acquired a cellular structure, and the strength of a natural connexion. The employment of the instrument was in no instance attended by severe pain, and the inflammation confined to the immediate neighbourhood of the parts inclosed. M. Dupuytren was the more surprized at this, as the instrument is not a simple cutting one, but produces a slough of the parts which it contains, which slough invariably separates between the blades. Satisfied with the results of his experiments, our author at length employed the enterotome in the case of a patient at the Hôtel Dieu.

*Case.* Ménage, æt. 26, was admitted into the hospital, in the month of January, 1816, with an artificial anus in the right groin. He had been subject, since infancy, to an inguinal hernia, which became strangulated about a year before his admission, and terminated in mortification of the gut. The operation was performed, and an artificial anus was established, through which the evacuations passed, generally about an hour and a half after a meal. Eight weeks after the operation, he was attacked with colic, and passed his evacuations per anum, which afterwards occasionally occurred, though at considerable intervals.

On admission, the artificial anus was at least half an inch in diameter, and bordered by irregular protrusions of the mucous membrane of the gut, whilst behind there appeared, on the least exertion, a hernia, which frequently gave rise to invagination of the intestine. The skin around was raw and sore, the sufferings of the man excessive, the smell intolerable. The patient being anxious for an operation, M. Dupuytren, after calming the irritation of the parts, began to search for the two extremities of the intestine, and the exact position of the partition between them. At length this was accomplished, the blades of the enterotome separately introduced, as high as they would go, into each portion of the gut, and joined together moderately close. No pain was felt, and, on the next day, the blades were tightened, when some colic was experienced. In a few days, the blades were somewhat moveable. On the sixth, there were slight evacuations per anum, and, on the eighth, the instrument fell off, the blades containing a membranous band, in which the tunics of the two parietes of the gut were recognized. The length of the band was twenty lines, and its breadth two, forming the measurement of the depth to which the blades had gone, and the extent of the partition which had been removed within them. From this period, the fæces passed by the natural anus, but the artificial one, though narrowed, still continued open, notwithstanding the use of pressure, bandaging, sticking plaster, and lunar caustic. At length the edges were pared off, and brought together by the twisted suture, whilst a particular instrument for making pressure was employed. At the expiration of four months, the patient was presented to the Faculty of Medicine, entirely cured.

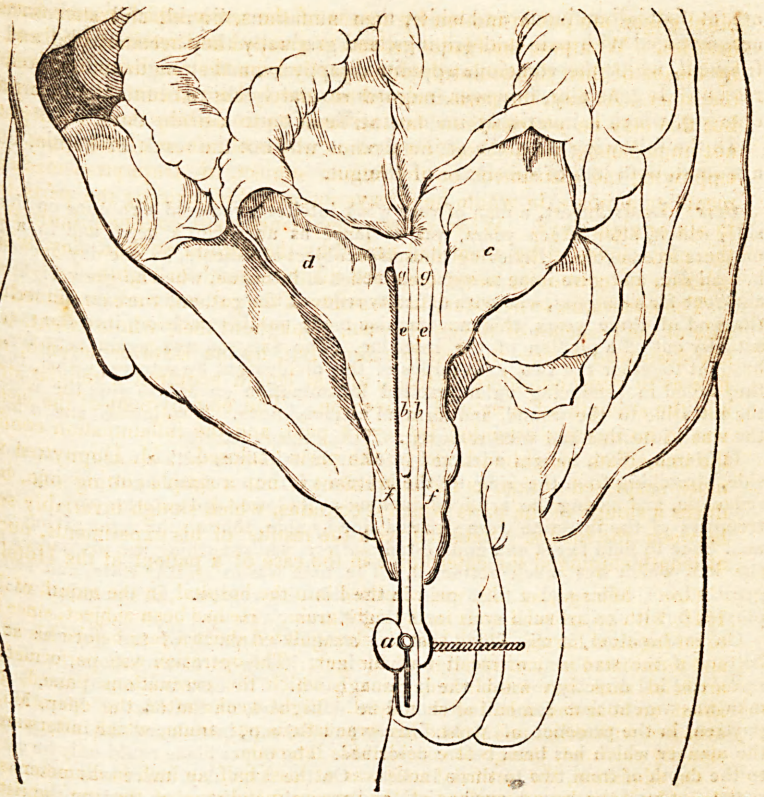

See Memoir.

A difficult part of the operation is to discover the lower orifice of the bowel, which requires that the finger or soft probe must be frequently employed for several days. This being ascertained, and the patient placed upon his back, one of the blades of the instrument is introduced, upon the index finger, into one of the orifices, to the depth of one, two, or three inches, according to the nature of the case. This blade is then given to an assistant, and the other introduced in the same manner into the other extremity of the gut, when they are joined together, like common forceps, by putting the tenon of the one into the mortise of the other. The partition is then inclosed within the blades, and, as their action should be slow and gradual, it is regulated by the screw. The pressure should destroy the life of the parts from the first day, as the pain and inflammation are thus prevented; it should also be increased every other day, by giving the screw a turn or two. Pain and colic are seldom experienced, at least to any great extent, and, after a few days, the instrument becomes a little moveable, the mobility increasing from day to day, until the enterotome at last falls off, which always happens between the seventh day and the tenth. The blades are nearly closed, and embrace within them the buttress and partition. Evacuations per anum sometimes precede the falling off of the instrument, are at first albuminous and white, then numerous, liquid, and stercoraceous, accompanied with pain and gripings, and gradually they become solid and less frequent, whilst the voracious appetite disappears, and strength and embonpoint return. It generally, however, requires several weeks to obliterate the artificial opening, which is invariably the most difficult portion of the cure.

In the following case, the artificial anus was not the result of hernia, but of a wound, with loss of substance of the gut.

*Case.* Louis Jubert, a man of very weak intellect, received a rupture on the left side, which, after fifteen years, was as large as an infant's head, and in a great measure irreducible. Believing himself an object of ridicule, he one day opened the hernial sac, and gave issue to eighteen inches of intestine, when he grew frightened, and sent for a surgeon, who with difficulty reduced the gut, and the case did well. At the end of three years, this monomaniac made another incision, into the sac, and actually cut off a portion of the intestine. The surgeon was again sent for, and brought together the two extremities of the divided gut by sutures, which, though they failed in producing union, excited inflammation enough to glue the edges of the intestine to the wound, and form an artificial anus. Two inches and a half of the small intestine had been removed.

In March, 1824, he was admitted into the Hôtel Dieu, with a tumour on the left side, stretching from the ring to the bottom of the scrotum, hard, shining, partly reducible, and having the artificial opening at its lower and anterior part. The extremities of the intestine were situated side by side, that on the right giving continual issue to fluid fæces and undigested matters, whilst nothing was discharged from the left, which was much retracted. The man was in a filthy state, and suffered greatly from colics and a fixed pain in the left iliac region. Compression was employed, but it excited such symptoms that it was given up.

On examination, it was found that the *stomach* extremity of the gut was at the bottom of the scrotum, where it formed inextricable circumvolutions, whilst the *rectal* end led directly towards the inguinal canal. The case was a bad one, but the man was urgent that something should be done, and, on the 31st of May, M. Dupuytren, in the presence of M.M. Larrey and Sanson, introduced the instrument in the manner which has been before described. The upper blade could only be carried to the depth of from two to three inches. On the next day, there was œdema and redness around the wound, on the sixth and seventh, slight colics, and, on the eighth, the instrument fell off. Clysters were daily given, and fetid gas, but nothing more, passed per anum. On the 14th day, the patient thought some fæces came away, and the volume of the tumour was diminished, soon after which violent pains were experienced in the belly, and the evacuations became established in the proper channel.

The volume of the tumour went on diminishing, but fæcal matter was still discharged through the artificial opening. To check this, an instrument was constructed, which produced very effectual pressure, but, as it sometimes fell off, and produced excoriation of the parts, it was removed, and a herniary bandage substituted in its stead. The patient now gained flesh and spirits, whilst the fæces were not only re-established by the natural passage, but the hernial tumour, which had been the cause of all his miseries, was returned into the abdomen. A triangular flap of skin, situated at the upper part of the artificial anus, was touched with a solution of lunar caustic, as well as the edges of the opening, and the two brought together by a proper bandage. Union took place, and the cure was completed in less than five months from the admission of the patient.

M. Dupuytren informs us, that twenty-one operations have been performed by himself with the enterotome, and twenty by other practitioners. Out of the forty-one patients thus operated on, three only died, one from effusion of fæcal matter into the abdomen, one from indigestion, and one from peritoneal inflammation. Of the thirty-eight remaining patients, the greater number experienced no bad symptoms whatever; twenty-nine were radically cured in from two to six months, and nine preserved, in spite of every thing, fistulous openings of greater or less extent, which obliged them to wear a bandage. Three-fourths of the anuses were the consequence of gangrene from strangulated hernia, and the other fourth of wounds, with loss of substance, in the intestine.

It appears, then, that the mortality of the operation is one in fourteen, and if we exclude the case of indigestion, which cannot fairly be considered a consequence of the operation at all, it is reduced to one in twenty.

We have thus, at considerable labour, presented our readers with an analysis of the above paper. In the original, it occupies nearly sixty pages, and we have compressed it, by means of a smaller type, and much abridgement, into ten or eleven. The memoir itself is interesting, the plan of treatment ingenious, and highly deserving of attention, as it promises, in many cases, to remove one of the most loathsome maladies that flesh is heir to.

---

### V.

*Observations on Hospital Gangrene, &c.* By J. Boggie, M.D. Surgeon to the Forces.

[Transactions of the Medico-Chirurgical Society of Edinburgh, Vol. III. Part I.]

Although names are conventional, and, in their etymology, frequently convey no idea of the things they were designed to express, it does not seldom occur that, by their misapplication, they either communicate one entirely, or partially, false. It would certainly be difficult to construct a vocabulary, every term of which would be an epitomized commentary upon the object to which it was appropriated; but we conceive that much improvement is required, and that much improvement could be made; and that, in those cases wherein it might prove impracticable to make names the etymological signs of things, it would at least be easy to select such appellations as would not mislead, when they could not instruct.

Hospital gangrene only denotes one variety of this disease, and a variety in its geography, not in its essence. But hospitals do not occupy the whole map of this affection; its blight visits different and distant localities, and no wound lies without the limits of its influence. Wherever its causes exist and operate, whether in the public lazar-house, or private home, its destructive properties will be seen and felt, and hospitals are merely the more frequent arenæ of its power, by being more frequently infested with the circumstances which favour its production.

We would, therefore, prefer the epithet *contagious gangrene*, as more expressive and not less correct. This designation conveys to us the leading feature of the disease; and, since the place of its occurrence is as accidental as the spots
